# Effects of bed net use, female size, and plant abundance on the first meal choice (blood vs sugar) of the malaria mosquito *Anopheles gambiae*

**DOI:** 10.1186/1475-2875-11-3

**Published:** 2012-01-04

**Authors:** Chris M Stone, Bryan T Jackson, Woodbridge A Foster

**Affiliations:** 1 Department of Entomology, Agricultural Science Center North, University of Kentucky, S-225, Lexington, KY 40546-0091, USA; 2 Department of Evolution, Ecology and Organismal Biology, The Ohio State University, 318 West 12th Avenue, Aronoff Laboratory, Columbus, OH 43210, USA

## Abstract

**Background:**

The purpose of this study was to determine whether the sugar-or-blood meal choice of *Anopheles gambiae *females one day after emergence is influenced by blood-host presence and accessibility, nectariferous plant abundance, and female size. This tested the hypothesis that the initial meal of female *An. gambiae *is sugar, even when a blood host is available throughout the night, and, if not, whether the use of a bed net diverts mosquitoes to sugar sources.

**Methods:**

Females and males <1-day post-emergence were released in a mesocosm. Overnight they had access to either one or six *Senna didymobotrya *plants. Simultaneously they had access to a human blood host, either for 8 h or for only 30 min at dusk and dawn (the remainder of the night being excluded by an untreated bed net). In a third situation, the blood host was not present. All mosquitoes were collected in the morning. Their wing lengths, an indicator of pre-meal energetic state, were measured, and their meal choice was determined by the presence of midgut blood and of fructose.

**Results:**

Female sugar feeding after emergence was facultative. When a blood host was accessible for 8 h per night, 92% contained blood, and only 3.7% contained sugar. Even with the use of a bed net, 78% managed to obtain a blood meal during the 30 min of accessibility at dusk or dawn, but 14% of females were now fructose-positive. In the absence of a blood host, and when either one or six plants were available, a total of 21.7% and 23.6% of females and 30.8% and 43.5% of males contained fructose, respectively. Feeding on both sugar and blood was more likely with bed net use and with greater plant abundance. Further, mosquitoes that fed on both resources were more often small and had taken a sugar meal earlier than the blood meal. The abundance of sugar hosts also affected the probability of sugar feeding by males and the amount of fructose obtained by both males and females.

**Conclusion:**

Even in an abundance of potential sugar sources, female *An. gambiae *appear to prefer a nearby human source of blood. However, the decision to take sugar was more likely if energy reserves were low. Results probably would differ if sugar hosts were more attractive or yielded larger sugar meals. The diversion of energetically deprived mosquitoes to sugar sources suggests a possible synergy between bed nets and sugar-based control methods.

## Background

The sub-Saharan malaria vector *Anopheles gambiae s.s*. uses two nutrient sources, plant sugar and vertebrate blood [1,2]. A female will take a relatively small number of large meals throughout its life, imparting every single feeding decision with importance [3]. But how the abundance of sugar-bearing plants and the accessibility of blood hosts in an environment affects the decision to feed on either sugar or blood is currently not well understood, even though it pertains to malaria epidemiology through its effects on vectorial capacity [4,5]. It also pertains to vector control through its implications for behaviour of mosquitoes around bed nets and for novel sugar-based control methods that exploit the sugar-feeding proclivity of mosquitoes. A promising example of such a method is the use of attractive toxic sugar baits (ATSB) that employ fruit scents to attract both male and female mosquitoes, a sucrose solution to stimulate feeding, and an oral insecticide [6]. This method is highly effective at controlling a variety of mosquito species in arid areas [7-9]. In an area in Mali, its use resulted in effective control of a population of *An. gambiae *[10], underscoring its importance to malaria control prospects.

In recent years access to insecticide-treated nets (ITNs) has increased greatly in sub-Saharan Africa, sufficient to cover an estimated 76% of persons at risk by 2010 [11], which has resulted in a decrease of malaria incidence in many countries. Perhaps the greatest threat to this progress is the selection for resistance to pyrethroid insecticides [12], and additional methods to manage the problem will become increasingly important [13]. If mosquitoes can be diverted from nets to sugar sources, the use of oral insecticide-laced sugar baits to manage resistance may be worth considering. Very little is known of the likelihood of feeding on sugar near a human covered by a bed net, save one report that mortality due to starvation in artificial huts could be minimized by placing glucose pads in the hut [14]. One related concern is behavioural resistance to bed nets, i.e., vectors respond to extensive bed-net coverage by changing their feeding behaviour, either by adaptation or inherent plasticity, by biting at times when people are not protected by nets or by exhibiting exophagy [15,16]. This type of resistance might be facilitated by increased feeding on sugar, if females make use of this resource to avoid starvation after being repelled from a house. Indeed, one of the great knowledge gaps of mosquito behaviour is what decisions females make after exiting a dwelling that houses a net, and how likely they are to be diverted to a non-human, to a different human, to nectar, or to a resting site. Yet, to reduce infection levels below that attainable by extensive treated-net coverage, it will be necessary to employ additional control methods that target repelled, exophagic, or early-biting mosquitoes [17]. Thus there is a need for novel control methods that are synergistic with ITNs [18,19]. A prerequisite to assessing the potential of sugar-based control methods to complement bed-net programmes is knowledge of whether mosquitoes are likely to obtain sugar after a failed attempt to feed on a human under a net.

How access to blood hosts and nectariferous plants affects the feeding decisions of *An*. *gambiae *will determine in which types of environment sugar-based control methods are likely to be effective. The feasibility of using ATSB stations or treated vegetation as stand-alone control efforts or as a part of integrated vector management [20] in a given area will depend on the frequency of sugar feeding by female anophelines in the multitude of sub-Saharan environments. This is true especially in more verdant areas, where these attracticides may have to compete with many natural sugar sources. Further, in environments where larval development sites are interspersed among human habitations or located nearby, sugar baits may face competition with blood hosts, as well. The relevance of the latter for a species such as *An. gambiae s.s*. will be directly related to whether newly emerged females are obligatory sugar feeders (which would imply a high vulnerability of this species to the method), or whether even at this age sugar constitutes a facultative part of the female diet, and blood may be taken instead.

*Anopheles gambiae *emerges from the aquatic pupal stage with energy reserves near a critical minimum, so that both sexes die within a few days without some form of adult nutrition [21]. Consequently, particularly in smaller females, the fate of the first meal differs from later (blood) meals in that it serves primarily in the synthesis of maternal reserves [21,22]. Adults live in close association with humans, often resting inside houses, and many populations use humans primarily or almost exclusively as their source of blood [23], a trait with unusual consequences. Human blood is low in one of the essential amino acids, isoleucine, thus limiting the amount of all amino acids derived from haemoglobin that can be converted into egg yolk protein. The excess amino acids are catabolized and make a large contribution to the energy reserve [21]. Some authors suggest that sugar is rarely or never taken by *An. gambiae *females [24-26], which makes sense in light of the energy derived from human blood and their easy access to it. However, by focusing on host-seeking females, researchers may have underestimated the proclivity of young females to sugar feed in order to increase their teneral reserves. Evidence from the field, not just laboratory and screenhouse experiments, demonstrates unequivocally that sugar feeding is a common feature in the lives of both sexes of this species [10,26,27] and H Manda, WA Foster, et al. in preparation.

State-dependent behavioural models developed to elucidate the decision to feed on sugar or on blood for female mosquitoes [28-30] provide a framework by which blood and sugar availability, and the risks associated with procuring each, can be factored in. These models make the assumption that plant sugar is a normal part of any mosquito's natural history and that, by extension, a sugar-or-blood decision is determined by three factors: internal state, a built-in assessment of costs associated with food availability, and the stimulus strength of each food. One can infer from the model of Ma and Roitberg [29] that *An. gambiae *females would choose to feed first on sugar when they are away from the blood-feeding habitat, but around domiciles the likelihood of sugar feeding declines sharply with increasing blood-host availability.

To date, experimental studies on the behaviour of one-day-old *An. gambiae *females support the notion that this feeding decision is opportunistic, but with some bias towards sugar. In an olfactometer, one-day-old females showed a higher response towards honey-baited ports than to ports baited with human-related volatiles (a soiled sock), and preferred honey when both were presented simultaneously, suggesting that sugar is a viable food choice for young females [31]. In a mesocosm containing both sugar-bearing plants and sucrose or honey solutions, where females had nightly access to a human, the majority of one-day-olds had taken a sugar meal, but a proportion had taken a blood meal instead [32]. And when sugar was absent, a greater proportion of females had taken a blood meal on their first night. This suggests that their initial meal choice is flexible and depends on the availability of each resource. But nothing is known of how the interacting factors determine the outcome of the mosquito decision-making process.

The approach to the sugar-blood decision described here, taken with a sense of immediacy for understanding a mosquito species of immense importance to human health, is a compromise between a field experiment and a laboratory one. This empirical study examined the influence of environmental conditions, i.e., the use of a bed net by a blood host and the abundance of nectar-bearing plants, as well as female size - an indicator of its energetic reserves - by creating several semi-natural conditions and observing a mosquito's decisions under those conditions. The main objectives were to see whether the initial meal of female *An. gambiae *favours sugar even when a blood host is available throughout the night, and, if not, whether the use of an untreated net is likely to divert mosquitoes to sugar sources.

## Methods

Mosquitoes (*Anopheles gambiae s.s*., Mbita strain) were reared according to standard methods, as previously described [33], with the following modifications: larvae were reared either at a high or low density to generate a wide size range of experimental mosquitoes. High-density pans held 300 larvae and received a food regime of 0.13 mg of finely ground Tetramin fish flakes per larva during each of the first three days of development, 0.26 mg for each of the next three days, and 0.53 mg for subsequent days until pupation. Low density pans held 50 larvae and received 0.4, 0.8, and 1.6 mg of food per larva for those same sets of days, respectively. On average, pupation occurred nine days after eggs were laid.

Pupae, approximately 200 from each rearing density, were placed in cups of water within small cages containing water wicks. Pupae were not separated by sex. On the day following synchronous nocturnal emergence the cages were placed in a large mesocosm within a glass greenhouse and the adults of both rearing densities released from them in the afternoon, approximately four hours before sunset. That night the mosquitoes would have access to differing levels of plant and blood hosts (depending on treatment, see below) upon which they could feed. Swarming at dusk has been observed in the mesocosm (CS, pers. obs.) and may have occurred during this study. However, in a previous study 1-day-old females were found to prioritize taking a blood or sugar meal before mating [32]. Although not assessed in this study, the assumption, therefore, was that the meal choice in this experiment was not influenced by female mating status. Both male and female mosquitoes were recovered from resting sites the following morning by backpack and mouth aspirators, between one and two hours after sunrise. They were killed and stored immediately at -40 C, to stop metabolic processes.

**Figure 1:**
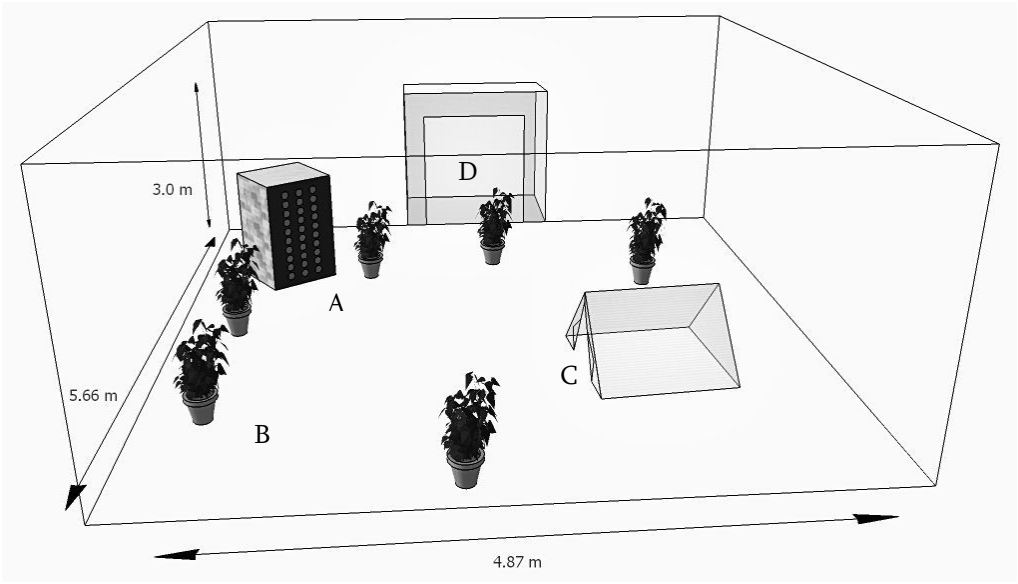
**Diagram of the mesocosm, with A) bank of resting sites, B) *Senna didymobotrya *plants, C) sleeping pad and mesh netting D) antechamber**.

The mesocosm (Jackson et al., in preparation), was a customized insect cage, 5.66 × 4.87 × 3.00 m (L × W × H) = 82.69 m^3^, enclosing a bank of mosquito resting sites, potted plants, and the inner vinyl-and-netting part of a two-person camping tent, which could be closed off (excluding mosquitoes) or opened (allowing mosquitoes access to a sleeping human) (Figure 1). Briefly, the resting sites structure (0.8 × 0.6 × 1.4 m) consisted of three concrete cinder block walls and one dark-stained wooden board in which 30 cardboard mailing tubes, painted black inside, were inserted [34]. The sides and ceiling of the mesocosm were made with white polyester netting, whereas the floor material was white vinyl. Nine 400-W metal-halide growing lights were suspended inside of the cage. These lights were on only between 08:00 and 17:00, so as not to interfere with the natural increase and decrease of light at dawn and dusk.

Temperature was controlled by the greenhouse heating and cooling systems. Wall-mounted steam radiators heated the room while louvered roof vents, an exhaust fan, and an evaporative cooling system maintained cooler temperatures, all of which were controlled by a thermostat within a sensor module suspended in the middle of the room ca. 1 m from the floor. Mean, mean minimum and mean maximum temperatures during experimental nights were 23.9°C ± 1.8, 22.4°C ± 2.0, and 27.6°C ± 2.9, respectively. Humidity was maintained with an industrial ultrasonic humidifier, which was controlled by a humidistat attached to the sensor module. Mean, mean minimum and mean maximum relative humidity during experimental nights were 66.3 ± 11.4, 55.2 ± 13.7, and 73.4 ± 12.2, respectively.

There were six treatment levels. These consisted of two levels of plant availability: either one, or six potted *Senna didymobotrya *(Fabaceae), a common plant in Kenya that *An. gambiae *readily feeds on, both in cages and in the field [35] and WAF unpubl. There were three levels of blood-host availability: 1) no blood host was present; 2) blood host available in early night and dawn; and 3) blood host available all night. In level 2, a human volunteer (CS) sat in the mesocosm with lower legs exposed and allowed mosquitoes to feed for the first half hour of full darkness (22:30 - 23:00) and he was exposed again at dawn (ranging from 06:30-07:00 to 07:30-08:00, so as to overlap with sunrise at 39°57' 40" northern latitude between mid-July and October). Between those exposures, he slept in the closed tent to simulate the use of an untreated but effective bed net. During the morning biting period, females were aspirated as they bit, so that body sizes of early-night biters (i.e., blood-fed females collected from the resting sites) and dawn biters could be compared. In level 3, a human volunteer (C.S.) slept in the open tent, exposed from 23:00-07:00, allowing mosquitoes to feed throughout the night. All were collected from resting sites in the morning. Four replicates were performed of each of the six plant-host abundance/blood-host availability permutations, a total of 24 overnight tests.

The following information was recorded for each subsample (total of all replicates: n = 3,167, 80-230 per night) of mosquitoes collected from biting catches and resting sites after sunrise: 1) sex; 2) body size (as indicated by wing length from alular notch to distal edge, excluding the fringe, measured by ocular micrometer; large = above median, small = below median [see results, section V]); 3) whether a resting female had taken a blood meal (determined by visual inspection); 4) whether a female was collected at dawn, while biting; and 5) sugar positivity and amount of fructose ingested (cold-anthrone method of Haramis and Foster [36]). Females were scored in these feeding categories: unfed, sugar-fed, blood-fed, or blood- and sugar-fed.

### Analysis

There are four possible outcomes of the feeding decisions made by females: they can 1) not feed on anything; 2) feed on sugar; 3) feed on blood, or 4) feed on both sugar and blood. These outcomes may reflect different processes. For instance, remaining unfed rather than feeding on either resource may have more to do with a mosquito failing to find any food than with choice, whereas taking a sugar or a blood meal implies a choice. To tease apart the decision components, these outcomes were analysed using generalized linear mixed models and linear mixed models in R [37], including replicate night as a random effect [38] (questions 1-5, below), or linear and logistic regression, including a term for replicate night (question 6, below), in JMP v9 (SAS Institute Inc., Cary, NC) to see which factors determined the following:

1 Whether a female mosquito fed on something (sugar and/or blood) or nothing. A priori expectations were these: with both increased plant- and blood-host availability, feeding on something becomes more likely; and larger females, having larger energy reserves, are more successful at locating food and feeding;

2 Whether a female mosquito fed on sugar or on blood. This required an analysis of a subset of the whole data set. Only females that fed on either sugar or on blood were considered, and the treatment where no blood host was available was not examined, because mosquitoes did not have a relevant food choice there. A priori expectations were these: sugar feeding increases with sugar availability, and blood feeding increases with blood-host availability. Due to their greater reserves, large females will prioritize blood feeding, whereas small females will prioritize sugar feeding to augment their limited reserves;

3 Whether a female fed on one resource (sugar or blood) or on two resources (sugar and blood) in the same night. Again a subset was analysed: the treatment without a blood host is not relevant, and females that did not feed at all were excluded. A priori expectations were these: feeding on both sugar and blood is most common when blood hosts are not accessible throughout the night, so that females are diverted to a sugar meal instead, with some of those then taking a blood meal at dawn;

The independent variables were as follows: 1) mean relative humidity and temperature, recorded on each experimental night; they were included because they might influence the activity of mosquitoes or nectar production; 2) body size (large or small), to see whether the energetic reserve affects the blood/sugar choice; 3) plant availability (two levels); and 4) blood-host availability (three levels, except two levels for analyses 2 and 3 - see above). All variables were initially entered into the model. The decision to include interactions was based on a graphical examination of the interaction profiles. Final models were attained using a backward selection procedure.

Further, the following questions were asked:

4 Does the probability of male sugar feeding depend on the abundance of the plants, male size, temperature, or humidity? Because males rely only on sugar, the answer provides more accurate insight into the effect of plant abundance on the likelihood of finding sugar;

5 Does the amount of fructose ingested by sugar-positive males and females depend on the independent variables mentioned? If so, this would indicate the possibility of competition for limited amounts of nectar;

6 Are there differences among females, in body size and the tendency to feed on both foods, between those biting in the early night and at dawn when blood-host access is restricted by a net? Both responsiveness to hosts [39] and persistence [40] might affect the outcome.

## Results

### What determines whether females feed on something (either blood or sugar) or nothing?

**Figure 2:**
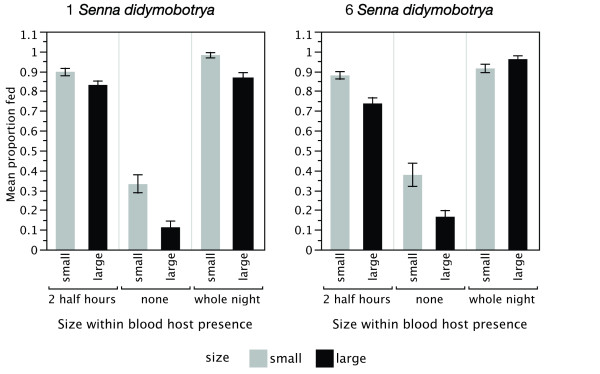
**Proportions ± SEM of small and large females that fed on at least one food item (i.e. blood or sugar or both) compared with nothing, according to blood-host exposure**. Both plant densities and all replicates are combined.

Both the size of females and their access to human blood affected whether females obtained a meal of either type, or remained unfed (Figure 2). Small females were more likely to have taken a meal than large females. The propensity to feed was significantly lower when no blood host was available, and there was a significant interaction between blood-host availability (none) and size, indicating that small females were more likely than large females to switch to sugar when no blood host was available (Table 1). An interaction between blood-host availability (whole night), plant abundance (six plants) and size was also significant: under these conditions the likelihood of small and large females to feed was reversed (Figure 2).

**Table 1:** Estimated coefficients, standard errors, z-values and p-values for the generalized linear mixed model for feeding or remaining unfed

Variable	**Coeff**.	se	z	*p *> z
Blood host (*none*)	3.99	0.63	6.32	2.55e-10 ***

Blood host (*whole night*)	-0.39	0.6	-0.65	0.51

Sugar host (*6*)	0.68	0.56	1.2	0.22

Size (*small*)	-0.56	0.25	-2.25	0.024 *

Blood host (*none*) * sugar host (*6*)	-1.45	0.86	-1.67	0.09

Blood host (*whole night*) * sugar host (*6*)	-2.16	0.92	-2.34	0.019 *

Blood host (*none*) * size (*small*)	-0.49	0.46	-2.05	0.039 *

Blood host (*whole night*) * size (*small*)	-1.44	0.81	-1.76	0.077

Sugar host (*6*) * size (*small*)	-0.28	0.35	-0.8	0.42

Blood host (*none*) * sugar host (*6*) * size (*small*)	0.75	0.63	1.2	0.22

Blood host (*whole night*) * sugar host (*6*) * size (*small*)	2.97	1.01	2.95	0.003 **

*Intercept*	-1.61	0.39	-4.06	4.81e-05 ***

*Replicate night*		0.73		

(n = 2141, log likelihood = -807.4)

### What determines whether a female chooses to feed on sugar or blood?

**Figure 3:**
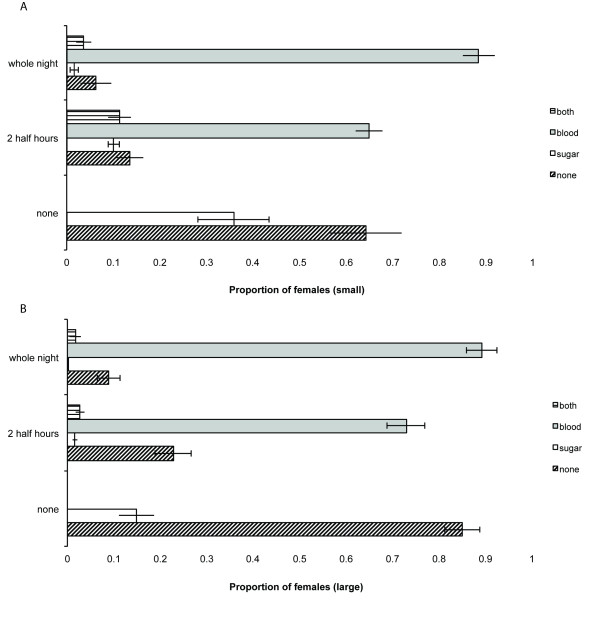
**a,b: The proportions of small (**a**) and large (**b) **females ± SEM that fed on blood, sugar, both, or neither, when a blood host was accessible for 8 h, or for 2.5 h, or was not present**. Both plant densities and all replicates are combined.

**Figure 4:**
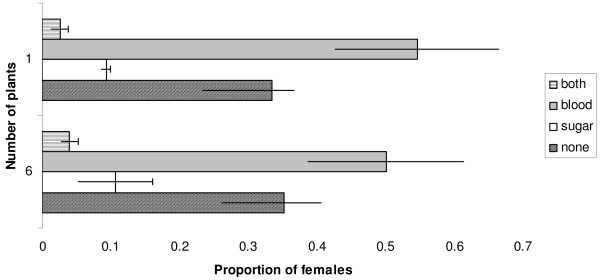
**The proportions ± SEM of females that fed on blood, sugar, both, or neither, when one or six *Senna didymobotrya *were present**. All replicates are combined.

A comparison of Figures 3 and 4 leads to the conclusion that the feeding choices of female *Anopheles gambiae *were determined to a great degree by the presence and accessibility of the blood host, and not by the abundance of potential nectar sources in the mesocosm. In a comparison of the sugar or blood choices by females when a blood host was present (restricted or unrestricted), the final model confirmed this observation, because it included blood-host availability (*z *= 4.69, *P *< 0.0001) as well as mosquito size (*z *= 5.34, *P *< 0.0001), but not plant abundance, temperature, or humidity, or any interactions among them. The standard error of the random intercept, replicate night, was minimal (0.000015).

### What determines whether a female feeds on just one or both resources?

Variables included in the model were blood-host availability, size, plant abundance, and mean temperature, and an interaction between plant abundance and blood-host availability (Table 2). Small females were significantly more likely to take both a sugar and a blood meal in one night than were larger females, and feeding on both foods was less likely when access to blood was unrestricted (Figure 3), in particular when only one plant was present.

**Table 2:** Estimated coefficients, standard errors, z-values and p-values for the generalized linear mixed model for feeding on one or two resources (sugar and blood)

Variable	**Coeff**.	se	z	*p *> z
Blood host (*whole night*)	3.04	1.02	2.96	0.003 **

Sugar host (*6*)	-1.33	0.61	-2.21	0.03 *

Size (*small*)	-1.61	0.26	-6.02	1.76e-09 ***

temperature	0.37	0.16	2.21	0.027 *

Blood host (*whole night*) * sugar host (*6*)	-2.27	1.08	-2.10	0.035 *

*Intercept*	-4.91	3.75	-1.31	0.19

*Replicate night*		0.16		

(n = 1496, Log likelihood = -334.4)

### Does the probability of male mosquitoes obtaining sugar depend on male size, plant abundance, temperature, or humidity?

**Figure 5:**
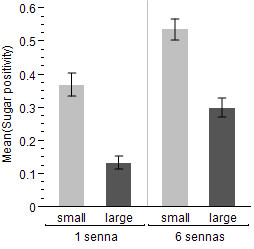
**Proportion ± SEM of large and small males that were positive for fructose after one night of exposure to either one or six *Senna didymobotrya***. All blood-host treatments, and all replicates, are combined.

Both sugar-host abundance (*z *= 2.41, *P *< 0.0001) and size (*χ*^2 ^= 8.41, *P *< 0.0001) had a significant effect on the proportion of males testing fructose-positive (Figure 5). With just one plant in the mesocosm, males were more likely to remain unfed than if six plants were present, and small males were more likely to be sugar-positive than large males. The standard error of the random intercept, replicate night, was 0.81.

### Amount of fructose

**Figure 6:**
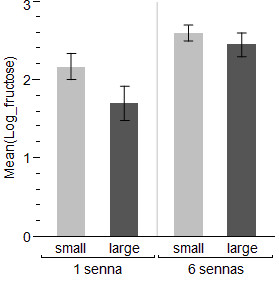
**Mean log amounts (μg) ± SEM of fructose per large or small male with one or six *Senna didymobotrya***. All blood-host treatments, and all replicates, are combined.

**Figure 7:**
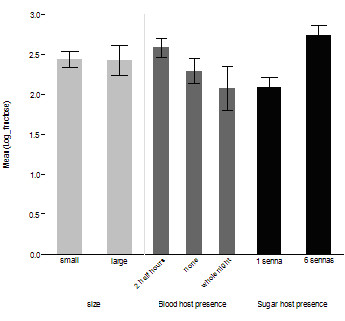
**Amounts of sugar (μg) ± SEM ingested by females according to size, availability of a blood host, and the number of plants present in the mesocosm**.

The final model on the size of the sugar meal taken by males included both the number of plants present in the mesocosm (*t *= 2.19, *P *< 0.05) and male size (*t *= 2.3, *P *< 0.05) (Figure 6). The variance of the random intercept, replicate night, was 0.16, and that of the residual term 1.61. The median wing length of experimental males was 2.88 mm (mean 2.88 ± 0.19 mm). The amount of sugar taken by females was greater when more plants were available in the mesocosm (*t *= 2.09, *P *< 0.05). Female size and the availability of a blood host in the mesocosm did not significantly affect the size of the sugar meal (Figure 7). The variance of the random intercept, replicate night, was 0.2, and that of the residual term 1.84. The median wing length of experimental females was 3.0 mm (mean 2.96 ± 0.24 mm).

### Differences between females taking blood in the evening and at dawn

Females biting in the evening had a mean wing length of 2.87 ± 0.26 mm and were significantly smaller than females biting at dawn, with a mean wing length of 2.96 ± 0.24 mm (*F*_1, 849 _= 13.9, *P *< 0.001). The term for replicate night was also significant (*F*_7, 849 _= 15.3, *P *< 0.0001). Overall, 44.2% of females that blood fed bit in the evening. Of the females that bit then, 6% were positive for fructose as well, whereas of those biting at dawn 12.5% were fructose-positive (*χ*^2 ^= 10.8, *P *= 0.001) when correcting for variation between replicate nights (*χ*^2 ^= 30.1, *P *< 0.0001), indicating that females that bit earlier in the night were less likely to obtain a sugar meal also. And of the females that bit at dawn, those that had obtained a sugar meal earlier were significantly smaller (wing length of 2.72 ± 0.22 mm) than those that were fructose negative (2.99 ± 0.23 mm) (*F*_1, 582 _= 80.7, *P *< 0.0001), with a term for replicate in the model (*F*_7, 582 _= 12.2, *P *< 0.0001).

## Discussion

The main results of this study were as follows: in a mesocosm with a sleeping human present, the majority of one-day-old females obtained a blood meal. This was the case even with bed-net use, though this did result in reduced numbers biting, corresponding to findings in the field [41,42]. When a blood host was not present, or access was restricted through the use of a net, sugar meals became more frequent, and smaller females were both more likely to take a sugar meal under these circumstances and more likely to take both sugar and blood on the same night. Plant abundance positively affected the likelihood of feeding on sugar in addition to blood for females, but not the choice of sugar or blood, in this experiment. Plant abundance also affected sugar positivity of males and the amount of fructose obtained by sugar-feeding mosquitoes.

The conclusion that plant abundance did not affect the choice for sugar or blood is tempered by two caveats. First, the availability of one nectar-producing plant, rather than six, may not be as important as the relative abundance of nectar-producing plants to non-sugar host plants. Second, if this plant species is not particularly attractive, or its nectar is difficult to locate (see more on this below), the proportion of females opting to take sugar is likely to be small, making detection of a significant difference less likely.

For the use of sugar baits as a malaria vector control tool, the strong tendency to feed on blood, even at one day post-emergence, suggests that in areas where larval-development sites are close to human habitations [43], the method may be useful mainly as a complement to bed nets. If larval development sites are located at considerable distance from humans, the dominance of blood feeding is a smaller issue. That is, though females are willing to feed on humans as early as 24 h after emergence, in nature they may not come into contact with humans that early, and attraction to sugar sources would be paramount.

While changes in biting times following the introduction of bed nets have been documented, these have been associated mostly with a shift in species [16,44]. Given the enormous selective pressure that presumably comes with broad ITN coverage, it is surprising that a shift in biting times of *An. gambiae s.s*. has not been more commonly observed [45], and this may be related to whether, in the field, behavioural avoidance is caused by selection or by plasticity. If a sufficient proportion of mosquitoes is repelled by nets, rather than killed outright, and females are likely to obtain sugar or blood elsewhere, one would not necessarily expect a change in host-seeking times to evolve. Flexibility was evident in this experiment, in which sugar feeding became more common when a bed net was used, warranting further studies on how the fitness costs of searching for an alternative host after being repelled is affected by the quality and abundance of plant hosts in the environment.

The response of females to plants in this study was considerably lower than in two earlier ones [31,32]. This cannot be explained entirely by the differences in blood-host availability, because in the present study even without a blood host, only approximately one third of females fed on sugar, while approximately half did so in smaller mesocosms, even when a blood host was present for 30 min per night [32]. Nectar meals are digested rapidly in this species (R.E. Gary, H. Manda, T.Guda, W.A. Foster, unpubl.) and small meals are undetectable even by cold-anthrone test after a few hours. The evidence from the field so far indicates sugar feeding in *An. gambiae *occurs mainly in the first part of the night, with a small peak at dawn [46]. Consequently, small sugar meals would have been digested by morning and would have tested negative for fructose, deflating the number of positives. An explanation for the low sugar rates is that the particular *S. didymobotrya *used here provided less sugar than sources used previously, which would have exacerbated the underestimation of nectar-feeding by the cold-anthrone test. Only a third of the males were fructose positive when only one plant was present, and the proportion of fructose-positive males, as well as the amount of fructose obtained by both males and females, increased when six plants were present, supporting the notion that nectar production was limited. For females, this level of sugar availability or plant stimuli may have been below a response threshold. Alternatively, it is possible that the difference in response to nectar, between this and previous studies, was due to the quality of the sources. Honey was present previously, and its volatiles may be more attractive. Yet, this plant ranked among the most "preferred" in experiments by Manda et al. [35], it is attractive to *An. gambiae *in olfactometers (M R Nikbakht Zadeh, pers. comm.), and it was present as a sugar source in a previous mesocosm experiment, in which it appeared to be favoured by male and female mosquitoes (CS, pers. obs.). Possibly, nectar production and attractiveness of this plant is condition-dependent, e.g., age, amount of new growth. Likewise, humans differ in their attractiveness to *An. gambiae *[47], and only one blood host was used in this study. Before the outcome of the current study can be extrapolated to the behaviour of mosquitoes in the field, further studies under semi-field conditions with a variety of plants and multiple blood hosts must be undertaken,

Small mosquitoes of both sexes were more likely than larger mosquitoes to have fed on the night after emergence. The opposite had occurred in an attraction study of *An. gambiae *on that night, about twice as many large-bodied mosquitoes of both sexes being attracted to honey as small ones [31]. The observed effect of body size in the current study may have been exaggerated by differential survival of mosquitoes from high- and low-density larval environments that failed to obtain a meal of either kind during their first full night as adults. But the mean wing length of females recovered when a blood host was absent or present for eight hours was 2.98 mm in both cases, and it was slightly lower (2.92 mm) when blood was restricted, a pattern that does not match the survival scenario. It makes sense that females with low energetic reserves should take sugar more readily than blood, being closer to starvation. Females with larger reserves may prefer not to feed on sugar and thereby keep their options open for a potential blood meal later that same night, a time frame within which a sugar meal may compete for abdominal space with a potential blood meal [48]. That result had been found for *Culex nigripalpus *[49], though in that case smaller females, despite more often choosing sugar, were less responsive to stimuli from both foods. For males, keeping their options open makes no sense. Alternative explanations for the present results are that the proportion of larger mosquitoes feeding on sugar may have been underestimated to a greater degree, if they exhibited greater flight activity, digested sugar faster than small mosquitoes, or tended to sugar-feed earlier in the night - these possibilities all require further research.

With the scaling-up of long-lasting insecticidal nets (LLIN) across sub-Saharan Africa since 2008 [11], one may question whether the behaviour of mosquitoes around untreated nets, as tested here, is relevant. With certain caveats, this mesocosm set-up is a useful proxy for studying bed-net related mosquito behavioural plasticity where LLIN coverage lags, or where some family members still use an untreated net [50], or insecticide efficacy has dissipated. Further, the behaviour around untreated nets can give insight into the behaviour of mosquitoes resistant to pyrethroids [12].

The decisions made in mesocosms provide clues to the behaviour of females after they are repelled from a house. *An. gambiae *often obtained blood at sunrise, after being frustrated a large part of the night. In the field, females may either continue seeking blood until an accessible host is found, or else cease host-seeking and possibly take a sugar meal, and then bite early the following evening. Such a shift to earlier biting, following the introduction of impregnated bed nets, has been reported in a number of studies [44,51] but has not been found in others [52]. Information on what happens to females repelled from houses is especially relevant to control in areas with a high degree of bed-net coverage, but no further reductions in parasitaemia [53]. In these cases, indoor control options will have to be supplemented with control methods targeting mosquitoes outdoors [54], for example by the use of zooprophylaxis, insecticide-treated resting sites, or larvicides. The present study suggests that males and small females are particularly likely to seek a sugar meal when access to blood hosts is restricted by bed nets, suggesting that a plant-based method may be an effective control tool for such endgame scenarios. The combination of sugar baits (for instance, placed indoors or near a house) and treated nets is one of these options. Its feasibility will require a bait that is substantially more attractive than the plant species used in this experiment, such as the one used in Mali [10]. Ideally, it should attract both sexes and all sizes, because males are essential to female fecundity [33], and large individuals may become more prominent as population numbers decline [55].

## Conclusions

The initial meal choice of a large majority of female *Anopheles gambiae s.s*. was human blood rather than plant sugar in these mesocosm experiments. Sugar feeding by 1-day-old females was thus facultative, i.e., dependent on environmental conditions, rather than obligatory. With reduced access to humans and reduced energetic reserves, these mosquitoes became more likely to feed on sugar. Thus, toxic sugar baits will be less effective if they have to compete directly with unprotected human hosts and natural sugar sources, unless these baits are very attractive. On the other hand, baits appear likely to work synergistically with bed nets. The diversion from protected humans to natural sugar sources may become an inevitable by-product of bed-net use and make the mosquitoes particularly vulnerable to toxic baits. Further studies require more natural circumstances and a greater variety of human hosts, plant hosts, and *Anopheles *species.

## Competing interests

The authors declare that they have no competing interests.

## Authors' contributions

All authors designed the mesocosm, which BTJ and CMS erected and set up for experiments. CMS conceived, performed, and analysed the experiments. All authors contributed to the writing of the manuscript and read and approved the final version.
